# Alcohol-Impaired Driving Among Adults — United States, 2012

**DOI:** 10.15585/mmwr.mm6430a2

**Published:** 2015-08-07

**Authors:** Amy Jewett, Ruth A. Shults, Tanima Banerjee, Gwen Bergen

**Affiliations:** 1 Division of Unintentional Injury Prevention, CDC; 2 University of Michigan Injury Center, Ann Arbor, Michigan

Alcohol-impaired driving crashes account for approximately one third of all crash fatalities in the United States (1). In 2013, 10,076 persons died in crashes in which at least one driver had a blood alcohol concentration (BAC) ≥0.08 grams per deciliter (g/dL), the legal limit for adult drivers in the United States (2). To estimate the prevalence, number of episodes, and annual rate of alcohol-impaired driving, CDC analyzed self-reported data from the 2012 Behavioral Risk Factor Surveillance System (BRFSS) survey. An estimated 4.2 million adults reported at least one alcohol-impaired driving episode in the preceding 30 days, resulting in an estimated 121 million episodes and a national rate of 505 episodes per 1,000 population annually. Alcohol-impaired driving rates varied by more than fourfold among states, and were highest in the Midwest U.S. Census region. Men accounted for 80% of episodes, with young men aged 21–34 years accounting for 32% of all episodes. Additionally, 85% of alcohol-impaired driving episodes were reported by persons who also reported binge drinking, and the 4% of the adult population who reported binge drinking at least four times per month accounted for 61% of all alcohol-impaired driving episodes. Effective strategies to reduce alcohol-impaired driving include publicized sobriety checkpoints (3), enforcement of 0.08 g/dL BAC laws (3), requiring alcohol ignition interlocks for everyone convicted of driving while intoxicated (3), and increasing alcohol taxes (4).

BRFSS is an ongoing, state-based, random-digit–dialed telephone survey that collects health risk data from noninstitutionalized adults aged ≥18 years (5). Data from the 2012 BRFSS survey were analyzed to estimate prevalence, number of episodes, and rate of alcohol-impaired driving by selected individual characteristics and rates by state and U.S. Census region. Data from all 50 states and the District of Columbia were included. In 2011, BRFSS began conducting interviews of respondents with mobile phones in addition to landline interviews (6). In 2012, approximately 78% of respondents completed the survey using a landline phone; response rates were 49% for landline and 35% for mobile phones (5), with 467,334 completed interviews. The 2012 BRFSS data were weighted using the raking method, which reduces the potential for bias (6). Respondents who reported consuming any alcoholic beverages within the past 30 days were then asked, “During the past 30 days, how many times have you driven when you’ve had perhaps too much to drink?”

Estimates of the annual number of alcohol-impaired driving episodes per respondent were calculated by multiplying the reported episodes during the preceding 30 days by 12. These numbers of episodes were summed to obtain state and national estimates of alcohol-impaired driving episodes. Annual rates of alcohol-impaired driving episodes were calculated by dividing the annual number of episodes by the respective weighted population estimate from BRFSS for 2012. For the 13 respondents who reported more than one episode daily, annualized alcohol-impaired driving episodes were truncated at 360. Rates were suppressed for five states because the number of episodes was <50 or the standard error was >30%.

Alcohol-impaired driving prevalence was stratified by sex and reported by age, race/ethnicity, education level, marital status, household income, number of binge drinking episodes, seat belt use (always wear or less than always wear) and U.S. Census region. Binge drinking was defined as women drinking four or more alcoholic beverages per occasion and men drinking five or more alcoholic beverages per occasion. Seat belt use among alcohol-impaired drivers was examined separately by type of state seat belt law. Primary enforcement seat belt laws (primary laws) permit law enforcement to stop motorists solely for being unbelted, whereas secondary laws permit ticketing unbelted motorists only if they are stopped for another reason (7). New Hampshire, the only state without a seatbelt law for adults, was included with the secondary law states. Differences between subgroups were analyzed using t-tests, with a p value of ≤0.05 indicating statistical significance.

In 2012, 1.8% of respondents reported at least one alcohol-impaired driving episode during the preceding 30 days. This represented 4.2 million adults who reported an estimated 121 million annual alcohol-impaired driving episodes, a rate of 505 per 1,000 population (Table 1). Among those who reported driving while impaired, 58% indicated one episode, 23% indicated two episodes, and 17% indicated 3–10 episodes in the past 30 days; 0.8% of respondents reported they drove while impaired at least daily. Men accounted for 80% of alcohol-impaired driving episodes. Young men aged 21–34 years, who represented 11% of the U.S. adult population, reported 32% of all episodes.

Persons who reported binge drinking accounted for 85% of alcohol-impaired driving episodes, and the 4% of the adult population who reported binge drinking at least four times per month accounted for 61% of all alcohol-impaired driving episodes. Persons who wore a seat belt less than always had an annual alcohol-impaired driving rate (1,321) three times higher than those who always wore a seat belt (398). Among alcohol-impaired drivers, those living in states with a secondary seat belt law were less likely to always wear their seat belt (55%) compared with those in states with a primary law (74%).

Annual alcohol-impaired driving episode rates varied more than fourfold among states, from 217 (Utah) to 995 (Hawaii) per 1,000 population (Table 2, Figure). The Midwest U.S. Census region had the highest annual alcohol-impaired driving rate at 573 per 1,000 population.

## Discussion

During 2012, an estimated 4.2 million U.S. adults reported driving while impaired by alcohol at least once in the preceding 30 days, resulting in an estimated 121 million alcohol-impaired driving episodes annually, and a national rate of 505 episodes per 1,000 population. Alcohol-impaired driving rates varied more than fourfold among states. Because BRFSS made changes in the survey weighting methodology and added a mobile telephone sampling frame since the alcohol-impaired driving question was last asked, direct comparisons of the 2012 results with those from earlier years were not possible. Nonetheless, the estimated number of alcohol-impaired driving episodes reported by U.S. adults in 2012 fell within the range of the 112 million to 161 million annual episodes reported from 1993 to 2010 (8). Also, young men aged 21–34 years and persons who binge drink have consistently reported the highest rates of alcohol-impaired driving. Likewise, persons living in the Midwest have consistently reported higher alcohol-impaired driving rates than those living in other regions.

Although reasons for the variation in alcohol-impaired driving across the United States are not fully understood, individual-level and state-level factors likely contribute. For example, in 2013, the estimated proportion of adults who consumed alcohol varied from 31% in Utah to 65% in Wisconsin (9). Additionally, effective prevention strategies have not been adopted by all states; for example, as of February 2015, 12 states prohibited the use of publicized sobriety checkpoints (10).

Seat belts are about 50% effective in preventing driver fatalities in crashes (1), and seat belt use is higher in states with a primary seat belt law compared with use in states with a secondary law (7). In this report, persons who did not always wear a seat belt had alcohol-impaired driving rates three times higher than those who were always belted. In addition, consistent seat belt use was especially low among alcohol-impaired drivers living in states with a secondary seat belt law. Taken together, these findings suggest that fatalities among alcohol-impaired drivers could be substantially reduced if every state had a primary seat belt law.

The findings in this report are subject to at least four limitations. First, self-reported alcohol-impaired driving as defined by the BRFSS survey cannot be equated to a specific BAC; however, 85% of episodes were reported by persons who also reported binge drinking. Second, because alcohol-impaired driving carries a stigma, these self-reported estimates might be underestimated because of social desirability bias. Third, BRFSS survey respondents were aged ≥18 years; therefore, alcohol-impaired driving episodes among younger drivers were not included. Finally, the median response rate for the 2012 BRFSS survey was only 45% (5), which increased the risk for response bias.

**Summary**

What is already known on this topic?

Alcohol-impaired driving crashes account for nearly one third of all motor vehicle crash fatalities.

What is added by this report?

In 2012, an estimated 4.2 million U.S. adults reported at least one episode of alcohol-impaired driving during the preceding 30 days, equating to an estimated 121 million annual alcohol-impaired driving episodes.

What are the implications for public health practice?

To reduce alcohol-impaired driving, states and communities could consider increasing the use of effective interventions such as publicized sobriety checkpoints, strictly enforcing 0.08 g/dL blood alcohol content laws and minimum legal drinking age laws, requiring ignition interlocks for all persons convicted of alcohol-impaired driving, and increasing alcohol taxes. To reduce alcohol-impaired driving fatalities, states and communities also might consider enacting primary enforcement seat belt laws.

Alcohol-impaired driving crashes have accounted for about one third of all U.S. crash fatalities in the past two decades (1,2). To reduce alcohol-impaired driving, states and communities could consider effective interventions, such as expanding the use of publicized sobriety checkpoints (10); enforcing 0.08 g/dL BAC laws and minimum legal drinking age laws (3); requiring ignition interlocks (i.e., breath-test devices connected to a vehicle’s ignition that require a driver to exhale into the device, and that prevent the engine from being started if the analyzed result exceeds a preprogrammed level) for all persons convicted of alcohol-impaired driving (3); and increasing alcohol taxes (4). Additionally, all states might consider enacting primary seat belt laws that cover all passengers to help reduce fatalities in alcohol-impaired driving crashes (7).

## Figures and Tables

**FIGURE:**
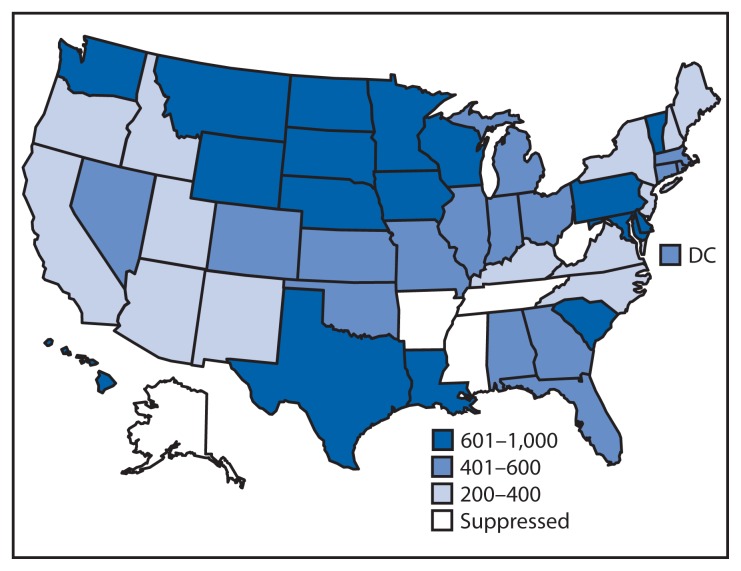
Annual rate* of self-reported alcohol-impaired driving episodes per 1,000 population, among adults — Behavioral Risk Factor Surveillance System, United States, 2012 **Abbreviation:** DC = District of Columbia. * Rates were suppressed if sample size was <50 or relative standard error was >30%.

**TABLE 1:** Percentage of adults reporting alcohol-impaired driving episodes during the preceding 30 days and annual rate of episodes per 1,000 population, by sex and selected characteristics — Behavioral Risk Factor Surveillance System, United States, 2012

Characteristic	Overall				Men				Women			
		
	%	No. of episodes	Annual rate	(95% CI)	%	No. of episodes	Annual rate	(95% CI)	%	No. of episodes	Annual rate	(95% CI)
**Total**	1.8	120,840,680	505	461–550	2.8	96,137,414	828	741–914	0.8	24,703,266	201	173–229
**Age group (yrs)**												
18–20	1.4	6,341,797	431	294–569	2.2	4,963,761	650	427–873	—*	—	—	—
21–24	4.2	16,709,636	1,004	814–1,195	5.8	12,301,238	1,450	1,113–1,787	2.6	4,408,397	540	373–708
25–34	3.0	32,662,609	794	630–958	4.5	26,597,672	1,282	962–1,602	1.5	6,064,937	297	240–355
35–54	1.9	44,360,681	527	450–605	3.0	35,183,421	844	700–988	0.9	9,177,260	216	158–274
≥55	0.8	20,631,892	252	210–295	1.4	16,987,417	453	365–541	0.3	3,644,475	82	56–108
**Race/Ethnicity**												
White, non–Hispanic	1.9	81,297,896	524	472–575	3.0	63,627,635	846	747–945	0.9	17,670,261	221	184–258
Black, non–Hispanic	1.8	12,262,181	440	349–531	2.7	8,901,599	698	528–869	1.0	3,360,582	222	137–308
Hispanic	1.8	18,638,930	518	363–673	2.9	16,579,282	917	611–1,223	0.6	2,059,648	115	78–152
Other, non–Hispanic	1.3	5,865,091	398	217–580	2.1	4,597,655	626	290–962	0.5	1,267,436	172	32–311
Multiracial, non-Hispanic	1.8	1,250,064	355	246–463	2.7	966,111	567	361–772	0.9	283,953	156	74–239
**Education**												
Less than high school	1.2	15,863,682	446	306–586	2.0	14,421,682	786	517–1,054	0.3	1,442,000	84	46–122
High school	1.6	33,534,025	486	422–551	2.6	27,365,716	792	676–907	0.6	6,168,309	179	120–239
Some college	2.0	42,280,497	578	472–684	3.3	33,526,025	1,012	788–1,237	1.0	8,754,472	219	162–275
College	2.2	29,162,476	474	426–522	3.2	20,823,990	691	607–775	1.3	8,338,485	266	219–313
**Marital status**												
Married	1.2	34,523,699	289	260–318	1.9	27,665,693	467	412–521	0.6	6,858,006	114	91–137
Unmarried couple	3.2	12,386,722	1,052	697–1,408	4.7	10,903,950	1,790	1,107–2,473	1.6	1,482,771	261	177–345
Previously married	1.6	24,538,321	521	422–619	3.0	18,620,065	1,051	811–1,291	0.7	5,918,256	201	138–265
Never married	3.0	48,329,111	798	670–927	4.2	37,973,371	1,155	930–1,379	1.6	10,355,740	374	284–465
**Annual household income ($)**												
<20,000	1.4	19,675,457	436	345–527	2.4	15,497,797	776	581–970	0.7	4,177,660	166	112–220
20,000–34,999	1.9	23,173,002	539	440–639	3.0	18,655,935	902	707–1,097	0.8	4,517,067	203	139–267
35,000–49,999	2.1	14,735,381	501	406–596	3.0	11,177,179	747	578–917	1.2	3,558,202	246	163–329
50,000–74,999	2.1	18,848,567	592	414–770	3.2	15,351,294	943	612–1,274	0.9	3,497,274	225	110–339
≥75,000	2.3	34,301,686	584	512–656	3.3	26,883,422	853	730–977	1.2	7,418,264	272	209–336
**Binge drinking**												
No binge drinking	0.8	14,753,474	181	158–204	1.2	10,177,543	253	211–296	0.5	4,575,932	111	91–131
1 time per month	4.7	11,359,118	840	690–989	5.5	8,213,096	1,027	791–1,263	3.6	3,146,022	569	440–698
2–3 times per month	8.2	19,039,754	1,611	1,388–1,834	9.7	13,917,849	1,832	1,566–2,097	5.5	5,121,905	1,213	812–1,614
≥4 times per month	14.8	73,285,148	5,637	4,875–6,398	16.2	61,905,024	6,520	5,519–7,522	11.0	11,380,124	3,244	2,453–4,035
**Seatbelt use**												
Less than always	4.0	42,356,829	1,321	1,101–1,541	5.3	36,527,500	1,843	1,497–2,190	2.0	5,829,329	477	344–609
Always	1.5	81,376,707	398	357–439	2.4	62,180,982	656	574–738	0.8	19,195,724	177	148–205

**Abbreviation:** CI = confidence interval.

* Sample size was <50 or relative standard error was >0.30.

**TABLE 2:** Annual rate of self-reported alcohol-impaired driving episodes per 1,000 population, among adults, by U.S. Census region and state — Behavioral Risk Factor Surveillance System, United States, 2012

U.S. Census region	State	Rate	(95% CI)
**National**		**505**	**(461–550)**
**Northeast**		**481**	**(389–572)**
	Vermont	881	(309–1,452)
	Pennsylvania	701	(409–992)
	Connecticut	558	(400–717)
	Rhode Island	522	(363–680)
	Massachusetts	510	(390–630)
	New York	372	(209–536)
	New Jersey	360*	(262–458)
	Maine	324	(172–476)
	New Hampshire	313*	(203–423)
**South**		**525**	**(433–616)**
	Louisiana	811	(463–1,159)
	Delaware	729	(429–1,028)
	Texas	703	(348–1,058)
	South Carolina	663	(346–980)
	Alabama	539	(241–837)
	Florida	539	(346–733)
	Maryland	527	(364–690)
	Georgia	491	(230–751)
	Oklahoma	467	(250–685)
	District of Columbia	409	(152–665)
	North Carolina	389	(253–525)
	Kentucky	388	(251–525)
	Virginia	308*	(206–409)
	Arkansas	—†	—
	Mississippi	—	—
	Tennessee	—	—
	West Virginia	—	—
**West**		**422**	**(351–493)**
	Hawaii	995§	(641–1,349)
	Montana	885§	(655–1,116)
	Wyoming	807	(342–1,272)
	Washington	706	(265–1,147)
	Nevada	489	(292–686)
	Colorado	477	(305–650)
	California	375	(273–477)
	Idaho	362	(122–602)
	Arizona	300*	(192–408)
	Oregon	285*	(168–402)
	New Mexico	273*	(180–367)
	Utah	217*	(98–337)
	Alaska	—	—
**Midwest**		**573**	**(498–649)**
	Nebraska	955§	(689–1,221)
	North Dakota	855	(473–1,238)
	Wisconsin	828	(536–1,121)
	South Dakota	733	(519–946)
	Iowa	715	(547–882)
	Minnesota	646	(457–835)
	Missouri	569	(294–843)
	Ohio	566	(415–716)
	Michigan	497	(326–667)
	Kansas	482	(335–629)
	Illinois	475	(223–727)
	Indiana	432	(224–639)

**Abbreviation:** CI = confidence interval.

* Significantly lower than the national rate.

† Sample size was <50 or relative standard error was >0.30.

§ Significantly higher than the national rate.

## References

[1] National Highway Traffic Safety Administration (2014). Traffic safety facts 2012: a compilation of motor vehicle crash data from the Fatality Analysis Reporting System and the General Estimates System.

[2] National Highway Traffic Safety Administration (2014). Traffic safety facts 2013: alcohol-impaired driving.

[3] The Guide to Community Preventive Services Motor vehicle-related injury prevention: reducing alcohol-impaired driving.

[4] The Guide to Community Preventive Services Preventing excessive alcohol consumption: increasing alcohol taxes.

[5] CDC Behavioral Risk Factor Surveillance System.

[6] CDC (2012). Methodologic changes in the Behavioral Risk Factor Surveillance System in 2011 and potential effects on prevalence estimates. MMWR Morb Mortal Wkly Rep.

[7] Highway Loss Data Institute, Insurance Institute for Highway Safety (2015). Safety belts.

[8] CDC (2011). Vital signs: alcohol-impaired driving among adults—United States, 2010. MMWR Morb Mortal Wkly Rep.

[9] CDC Behavioral Risk Factor Surveillance System. Prevalence and trends data: alcohol consumption 2013.

[10] Governors Highway Safety Association (2015). Sobriety checkpoint laws.

